# Supplementary figures and images for: Assessment of immunogenicity and protection induced by COBRA HA vaccines formulated with Infectimune in young and elderly ferrets
Source: PLoS One. 2026 Feb 19;21(2):e0339613. doi: 10.1371/journal.pone.0339613 (PMC12919834; doi:10.1371/journal.pone.0339613)

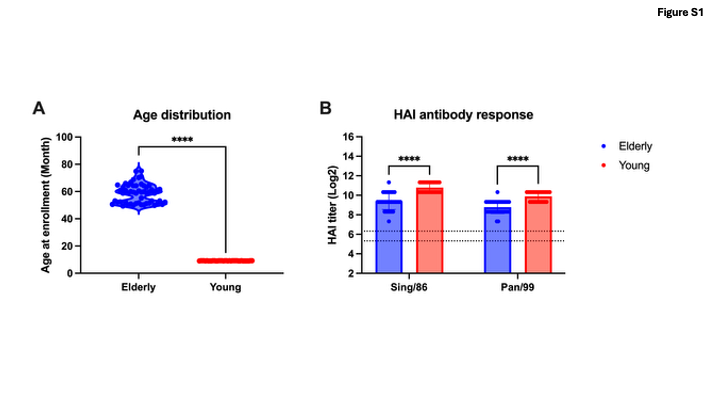

Supplement: S1 Fig — (A) The age distribution of young and elderly ferrets at the enrollment. (B) HAI antibody response after pre-immune infection. Individual ferret serum collected 2 weeks after pre-immune infections were used in HAI assay against SG/86 H1N1 and PN/99 H3N2 influenza viruses. The y-axis indicates HAI titer in Log2. The lower dashed line indicates 1:40 and the higher dashed line indicates 1:80. Data is presented as average ± standard deviation. Age data and HAI titers were statistically analyzed using one-way analysis of variance (ANOVA) by Prism 10 software. A P value of less than 0.05 was defined as statistically significant (*, P < 0.05; **, P < 0.01; ***, P < 0.001; ****, P < 0.0001). (TIFF) [file pone.0339613.s001.tiff]
